# N-of-1 Trials of Antimicrobial Stewardship Interventions to Optimize Antibiotic Prescribing for Upper Respiratory Tract Infection in Emergency Departments: Protocol for a Quasi-Experimental Study

**DOI:** 10.2196/50417

**Published:** 2024-02-21

**Authors:** Hersh Attal, Zhilian Huang, Win Sen Kuan, Yanyi Weng, Hann Yee Tan, Eillyne Seow, Li Lee Peng, Hoon Chin Lim, Angela Chow

**Affiliations:** 1 Accident & Emergency Department Changi General Hospital Singapore Singapore; 2 Department of Preventive and Population Medicine Office of Clinical Epidemiology, Analytics, and Knowledge Tan Tock Seng Hospital Singapore Singapore; 3 Department of Emergency Medicine National University Hospital Singapore Singapore; 4 Department of Surgery Yong Loo Lin School of Medicine National University of Singapore Singapore Singapore; 5 Department of Emergency Medicine Tan Tock Seng Hospital Singapore Singapore; 6 Acute and Emergency Care Department Khoo Teck Puat Hospital Singapore Singapore; 7 Saw Swee Hock School of Public Health National University of Singapore Singapore Singapore; 8 Lee Kong Chian School of Medicine Nanyang Technological University Singapore Singapore

**Keywords:** antibiotic resistance, emergency department, upper respiratory tract infection, N-of-1 trials, prescribing feedback, feedback, emergency, upper respiratory tract, respiratory, antibiotic, antimicrobial stewardship, antimicrobials, antibiotics, hospital, experimental study, antibiotic therapy, URTI, evidence-based intervention, evidence-based, patient education, prescribing rates, patient literacy, Singapore, regression analysis, regression

## Abstract

**Background:**

Antimicrobial stewardship programs attempting to optimize antibiotic therapy and clinical outcomes mainly focus on inpatient and outpatient settings. The lack of antimicrobial stewardship program studies in the emergency department (ED) represents a gap in tackling the problem of antimicrobial resistance as EDs treat a substantial number of upper respiratory tract infection cases throughout the year.

**Objective:**

We intend to implement two evidence-based interventions: (1) patient education and (2) providing physician feedback on their prescribing rates. We will incorporate evidence from a literature review and contextualizing the interventions based on findings from a local qualitative study.

**Methods:**

Our study uses a quasi-experimental design to evaluate the effects of interventions over time in the EDs of 4 public hospitals in Singapore. We will include an initial control period of 18 months. In the next 6 months, we will randomize 2 EDs to receive 1 intervention (ie, patient education) and the other 2 EDs to receive the alternative intervention (ie, physician feedback). All EDs will receive the second intervention in the subsequent 6 months on top of the ongoing intervention. Data will be collected for another 6 months to assess the persistence of the intervention effects. The information leaflets will be handed to patients at the EDs before they consult with the physician, while feedback to individual physicians by senior doctors is in the form of electronic text messages. The feedback will contain the physicians’ antibiotic prescribing rate compared with the departments’ overall antibiotic prescribing rate and a bite-size message on good antibiotic prescribing practices.

**Results:**

We will analyze the data using segmented regression with difference-in-difference estimation to account for concurrent cluster comparisons.

**Conclusions:**

Our proposed study assesses the effectiveness of evidence-based, context-specific interventions to optimize antibiotic prescribing in EDs. These interventions are aligned with Singapore’s national effort to tackle antimicrobial resistance and can be scaled up if successful.

**Trial Registration:**

ClinicalTrials.gov NCT05451863; https://clinicaltrials.gov/study/NCT05451836

**International Registered Report Identifier (IRRID):**

DERR1-10.2196/50417

## Introduction

### Background

The discovery of antibiotics is among the most important public health achievements in the 20th century [1]. However, inappropriate antibiotic use has driven a rapid increase in antibiotic resistance, and with the drying up of the pipeline of new antibiotics, a postantibiotic era is imminent [2-4]. Hence, there is a need to reduce antibiotic use by changing physicians’ antibiotic prescribing practices and reducing patient demand through antimicrobial stewardship programs (ASPs).

Despite the presence of ASPs in various settings, their effects are modest, as antibiotic prescribing practices have been difficult to change [5]. Systematic reviews of interventions to improve physicians’ antibiotic prescribing practices have suggested that the traditional isolated forms of physician education (eg, didactic presentations or printed education materials alone) have only produced slight improvements in prescribing practices [6,7]. However, interventions such as educational outreach or training of local opinion leaders [8,9], as well as behavioral “nudges” in the form of public commitment devices [10] and peer comparison [11], were more effective in improving physician prescribing behaviors. In a systematic review evaluating information leaflets on common infections in general practice, 3 out of 4 studies on antibiotic use showed significant reductions in antibiotic prescriptions [12]. Another study using patient education in the form of an information leaflet regarding antibiotics for bronchitis showed a significant reduction (49% vs 63%) in antibiotic use compared with the control group [13].

### Rationale for the Study

There is a need for more studies to assess the effectiveness of ASP in emergency departments (EDs) [14]. Antibiotics are often prescribed for upper respiratory tract infection (URTI) in the ED, although the routine use of antibiotics for URTI is not recommended [15,16]. A mixed methods study involving 8 EDs in 3 cities in the United States found that the fast-paced environment of the ED encouraged unnecessary antibiotic use [17]. In Singapore, URTIs also accounted for a substantial proportion of attendances at EDs and were associated with frequent ED attendance, as EDs were popular choices for primary care [18]. A previous study at an adult general hospital reported that 35% of adult patients presenting to the ED for URTI were prescribed antibiotics [19].

A physician’s likelihood to prescribe antibiotics for a patient is complex and determined by many factors. The determinants of antibiotic prescribing may include the physician’s worry about complications arising in the patient [20], busy clinical practice [17], insufficient physicians’ knowledge [21], underestimation of the effect of inappropriate antibiotic prescribing leading to antimicrobial resistance (AMR) [22], and perception of the patient’s expectations for an antibiotic [19]. Furthermore, decision fatigue can reduce the physician’s ability to resist ordering inappropriate treatments and increase inappropriate antibiotic prescribing [23].

Given the multifactorial nature of inappropriate antibiotic prescribing, a single approach will unlikely work for all physicians [24]. Furthermore, different patient populations may warrant a variety of interventions. Hence, there is a need for interventions tailored to physicians and patients to promote the judicious use of antibiotics. We describe the protocol of a quasi-experimental study on 2 antibiotic stewardship interventions in the ED according to the SPIRIT (Standard Protocol Items: Recommendations for International Trials) checklist [25].

### Hypothesis and Objectives

We hypothesize that (1) patient education via tailored information leaflets (addressing knowledge, perception, and belief gaps of the local patient population on antibiotic use for URTI) distributed while waiting for consultation with the physician can improve patient knowledge and change patients’ expectations for antibiotics. The change in patients’ expectations of antibiotics would reduce unnecessary antibiotic prescribing by physicians in response to patient demand in time-pressured EDs. (2) Surveillance of antibiotic prescribing rates and physician feedback enables physicians to reflect on their prescribing practices. Physician feedback by senior ED physicians, coupled with education on good antibiotic prescribing practices, serves as a reminder for physicians to improve their antibiotic prescribing practices.

Hence, we aim to evaluate the effectiveness of 2 tailored antimicrobial stewardship interventions in optimizing antibiotic prescribing for uncomplicated URTI cases in 4 adult EDs in Singapore. The two interventions are (1) patient information leaflets on appropriate antibiotic use and AMR. The ED triage nurses will provide pamphlets to patients suspected of URTI prior to their consultation with the physician. The pamphlets are available in Singapore’s 4 official languages. (2) Feedback to physicians on their antibiotic prescribing rates. A senior ED doctor will send a personalized SMS text message to each physician who has seen at least 1 patient in the ED in the previous month at 2 monthly intervals. The feedback will contain the physicians’ antibiotic prescribing rate compared with the departments’ antibiotic prescribing rate and a bite-size message on good antibiotic prescribing practices.

## Methods

### Study Design

**Figure 1:**
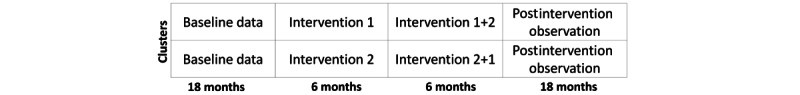
Quasi-experimental study of 2 tailored antimicrobial stewardship interventions in 4 emergency departments.

Our study uses a quasi-experimental design to evaluate the effects of interventions over time. The entire study period is from January 2021 to December 2023. All 4 EDs will be exposed to 2 interventions over 12 months, with the introduction of each intervention in a 6-month interval. The study will include an initial control period of 18 months, with none of the 4 hospitals exposed to any intervention. In the first 6 months, we will randomly assign 2 EDs to receive 1 intervention (ie, patient education), while the other 2 will receive the second intervention (ie, physician feedback). All EDs will subsequently receive the other intervention in the subsequent 6 months on top of the ongoing intervention. Data will be collected for another 6 months to assess the persistence of the intervention effects (Figure 1).

### Study Population

The study population includes physicians working in the adult EDs of 4 public hospitals in Singapore.

### Intervention

We will implement 2 interventions tailored to the context of EDs in Singapore. The interventions were selected based on a literature review of the effectiveness of ASP [12,26] and designed with input from ED physicians within our population via a qualitative study. We consulted senior ED physicians from our study sites to adjust the intervention to the local ED context and to facilitate buy-in prior to study implementation.

### Patient Education

Patient information leaflets were designed to educate patients on appropriate antibiotic use and AMR. The design of the leaflets was based on the information from the United States Centers for Disease Control and Prevention patient educational materials [27] and tailored to the local ED context.

Patient leaflets (Multimedia Appendix 1) were made available at the EDs of the participating sites. ED triage nurses identified patients presenting to the ED with URTI symptoms and provided these patients with the leaflet prior to their consultation with the physician. The leaflets were made available in Singapore’s 4 official languages—English, Chinese, Malay, and Tamil.

### Feedback to Individual ED Physicians by Senior Doctors

A senior ED physician in the department will send the feedback messages to the ED physicians in the institution. The feedback message will contain the physicians’ personal antibiotic prescribing rate and the department’s average prescribing rate in the past month and will be administered at 2 monthly intervals. Bite-sized information on tips to reduce antibiotic prescribing for URTI, obtained from evidence-based sources and adjusted based on inputs from senior ED physicians of our study sites, will be sent together with the personalized message. The feedback will be delivered via Tiger Text, a messaging platform used by employees of Singapore’s public health care institutions.

An example of the message is shown in Textbox 1.

**Example of a personalized physician feedback message.**

Dear Dr X,

 I would like to share with you the following feedback regarding antibiotic prescribing rates for URTI in TTSH ED.

 Last month, 200 patients that visited TTSH ED were diagnosed with URTI (primary or secondary diagnosis) and discharged from the ED. Among these patients, 100 (50%) were prescribed with antibiotics.

You were the primary physician of 10 patients and 5 (50%) of them were prescribed with antibiotics.

 I hope this feedback would be useful for your practice. Thank you for being our antibiotic guardian!

(Note: Common cold or non-specific upper respiratory tract infection (prominent symptoms include fever, cough, rhinorrhoea, nasal congestion, postnasal drip, sore throat, headache, and myalgia) are caused by at least 200 types of viruses. The receipt of antibiotics for common cold does not reduce the duration of symptoms.)

The medication and visit records of patients diagnosed with uncomplicated URTI will be extracted from the ED’s electronic medical records over 36 months (18 months before and 18 months after the intervention). Patient particulars were deidentified prior to data processing in the R software (R Foundation for Statistical Computing). The medications given to these patients will be identified, and their primary physicians will be given feedback on the antibiotic prescribing rates for the patients they have seen.

Uncomplicated URTI includes but is not limited to acute nasopharyngitis and acute URTI of multiple or unspecified sites (International Classification of Diseases, 9th Revision, Clinical Modification [ICD-9-CM] 460 and 465); acute pharyngitis, tonsillitis, laryngitis, and tracheitis (ICD-9-CM 462, and 463, and 464); and acute or unspecified bronchitis and bronchiolitis (ICD-9-CM 466 and 490). The listed ICD-9-CM codes are for acute respiratory tract infection diagnoses for which antibiotics are not usually indicated [28].

### Outcome Measure

The primary outcome measure of interest is physicians’ monthly antibiotic prescribing rate. The antibiotic prescribing rate will be computed by dividing the number of patients prescribed antibiotics by the total number of patients with URTI seen by the physician in the month.

### Sample Size

At the end of 12 months, all hospitals will be exposed to both interventions. Assuming 80 physicians per ED and an average of 1200 patients with URTI per ED over a 6-month period, a total of 640 physicians and 9600 patients will be exposed to the interventions in the 12-month intervention period.

### Statistical Analysis

We will analyze the data using segmented regression models. Segmented regression models allow adjustments to confounding factor and slope changes at various time points but do not account for cluster differences. The inclusion of cluster differences will increase the robustness of the model [29]. The equation of the model is shown as follows:

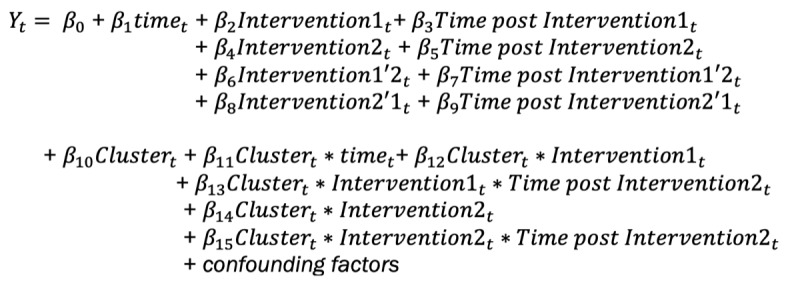



β1 measures the preintervention slope, β_2,4,6,8_ measures the immediate change in mean outcome from the previous level, while β_3,5,7,9_ measures the difference in slopes pre-post compared with the previous level. β_11_ measures the differences in groups or clusters at the preintervention level, β_12,14_ measures the differences in groups or clusters on changes at the start of the intervention, while β_13,15_ measures the differences in groups or clusters on changes in slope due to the intervention. Statistical software such as SPSS (IBM Corp), STATA (StataCorp), and R will be used for analysis.

### Ethical Considerations

This study was approved by the National Healthcare Group Domain Specific Review Board in Singapore (2019/00174). Informed consent was waived by the review board as all individual-level data were extracted from the electronic medical records and deidentified by the respective institutions’ independent trusted third parties. Data extraction does not interfere with the standard care the patients receive and does not pose more than minimal risk to the participants. No compensation is provided to the participants and the data team does not collect more data than required.

## Results

The results are expected to be achieved by January 2024. The data analysis and manuscript are expected to be completed by the end of 2024. The results will be presented at scientific meetings and published in international peer-reviewed journals.

## Discussion

### Principal Findings

Our protocol describes the design and implementation of a context-based, 2-pronged approach to tackle the issue of antibiotic misuse and overuse in the ED setting. Many ASPs have had limited success due to the lack of vital personnel involvement (ie, senior physician), misalignment with the local context, and lack of physician involvement [30]. We plan to have senior physicians deliver the feedback messages to increase physicians’ receptivity to the messages.

Hence, we designed the intervention with a rigorous review of evidence from the literature and considered the factors that contributed to the success of ASP. Our interventions were also tailored to the local ED context by considering the inputs of ED physicians via qualitative interviews and obtaining buy-in from senior physicians from the EDs.

Although randomized controlled trials are the most robust way of evaluating interventions, this approach is often not feasible in the health service setting in terms of blinding participants from interventions and preventing cross-contamination between the control and intervention groups. Quasi-experimental designs serve as a more practical option to evaluate health service interventions without compromising data robustness. Therefore, this study will provide an excellent opportunity for us to assess the effectiveness of a large-scale, context-based ASP in the ED.

With established processes already in place to store and distribute the pamphlets in the EDs involved in this study, we have received buy-in and will be able to re-engage the heads of departments of these EDs to continue our interventions or expand to other EDs in the long term if the intervention is shown to be effective.

### Potential Limitations

The antibiotic prescribing feedback may not be accurate if the primary physician is not the prescribing physician. Furthermore, patients attending the ED may have multiple complaints and may receive antibiotics for conditions unrelated to URTI. Hence, the feedback message had to be adjusted to reflect an accurate interpretation of the data. There may also be a lack of continuity of feedback should junior physicians rotate out of the ED. The lack of feedback continuity may affect the statistical effectiveness of feedback intervention, although physicians who received the intervention may have positive spillover effects to other departments upon rotating out of the ED.

Some patients may also miss the pamphlets when the triage nurses are overburdened with a high patient load and forget to hand out the pamphlets to them. We have placed the pamphlets in the ED waiting areas for patients to pick up the pamphlets should they be interested in the topic. The ED nurses will help to perform periodic checks to replenish the pamphlets when the stock is low.

### Conclusions

The lack of ASP in the ED represents a missed opportunity in a collective effort to tackle AMR nationally. Our protocol described a study that assesses the effectiveness of evidence-based, context-specific interventions to optimize antibiotic prescribing in EDs. These interventions are aligned with Singapore’s national effort to tackle AMR and can be scaled up to other EDs if successful.

## Appendix

Multimedia Appendix 1 English version of patient leaflet.

## Abbreviations

AMR: antimicrobial resistance
ASP: antimicrobial stewardship program
ED: emergency department
ICD-9-CM: International Classification of Diseases, 9th Revision, Clinical Modification
SPIRIT: Standard Protocol Items: Recommendations for International Trials
URTI: upper respiratory tract infection

## Data Availability

Data sharing not applicable to this article as no data sets were generated or analyzed for this protocol.
