# Supplementary material for: N-of-1 Trials of Antimicrobial Stewardship Interventions to Optimize Antibiotic Prescribing for Upper Respiratory Tract Infection in Emergency Departments: Protocol for a Quasi-Experimental Study
Source: JMIR Res Protoc. 2024 Feb 21;13:e50417. doi: 10.2196/50417 (PMC10918537; doi:10.2196/50417)
Supplement: Multimedia Appendix 1 [file resprot_v13i1e50417_app1.pdf]

Managing Your Illness

Home Rest

- Stay at home to rest for your body to recover.
- Avoid going to school, work, and crowded places.
- Do not engage in strenuous physical activity.

Tissue and Masks

- Use a tissue when you cough or sneeze and always wear a mask properly.
- Dispose the used tissues and masks in rubbish bins, and wash/sanitize your hands properly.

Frequent Hand Washing/Sanitization

- Wash your hands frequently with soap and water or rub your hands with alcohol sanitizers. This prevents you from spreading your germs to others.

<u>Disclaimer:</u>

This patient education leaflet is designed for the sole purpose of research, and is approved by the National Healthcare Group Domain Specific Review Board.

It is not meant for commercial distribution.

The information provided is accurate at the time of printing.

For more information on antibiotic use and antimicrobial resistance, please refer to the Health Promotion Board's website:

https://www.healthhub.sg/programmes/146/use-antibiotics-right

Patient Education Leaflet, English version 1.0, 30 May 2022

Good Antibiotic Habits: 4 Things to Know

Take antibiotics exactly as your doctor tells you to.

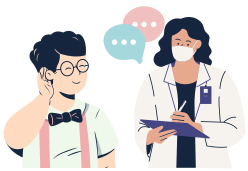

Do NOT take antibiotics prescribed for someone else.

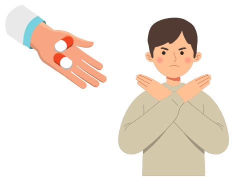

Do NOT save antibiotics for future use.

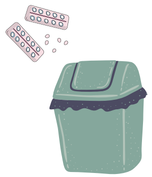

Do NOT share your antibiotics with others.

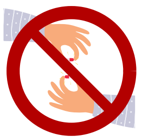

Information adapted from the U.S. Centers for Disease Control and Prevention: Antibiotic Do's & Don'ts

Antibiotics are NOT needed for infections caused by viruses

Antibiotics are used to treat infections caused by bacteria, but do not work on infections caused by viruses. Examples of infections caused by bacteria are urinary tract infections, skin infections and tuberculosis.

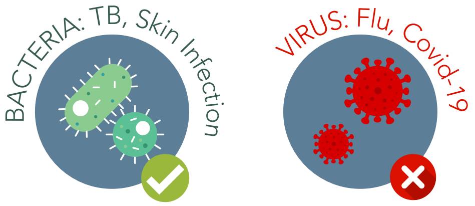

Examples of infections caused by viruses are the common cold, flu, and COVID-19.

Antibiotics are not necessary for treating the common cold, flu or COVID-19.

Recommendation:

Follow your doctor's advice and take the prescribed medication(s) as instructed.

Do NOT request for antibiotics from your doctor

Not all illnesses are the same, even if you have the same symptoms.

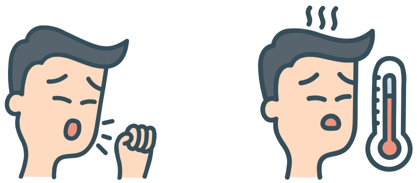

You do NOT need antibiotics for infections caused by viruses.

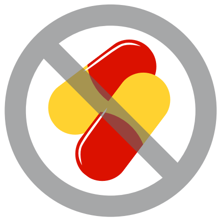

Recommendation:

Do NOT expect or request for antibiotics; your doctor will prescribe them if you need them.

Unnecessary use of antibiotics can be harmful

Unnecessary use of antibiotics causes antibiotic resistance

Antibiotic resistance occurs when the bacteria defeat the antibiotic that was designed to kill them.

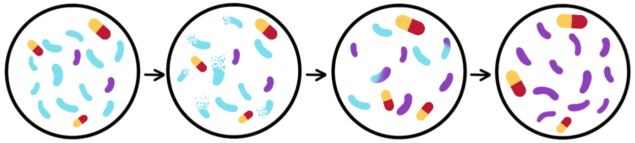

Legend:

- Antibiotic-susceptible bacteria (bacteria CAN killed by the antibiotic)
- Antibiotic-resistant bacteria (bacteria CANNOT killed by the antibiotic)
- Bacteria slowly becoming resistant to the antibiotic
- Bacteria being killed by the antibiotic
- Antibiotic

Antibiotic-resistant infections can happen to anyone.

A person who has antibiotic resistance may:

- take longer to recover from an infection
- have a higher risk when undergoing surgical procedures; and
- require more expensive and intensive treatments.

Recommendation:

Follow the doctor's advice on your treatment.
